# Systematic Comparison of the TGF-β Isoforms in Normal Dermal and Lung Fibroblasts Identifies TGF-β2 and TGF-β3 as Priority Targets in Tissue Fibrosis

**DOI:** 10.3390/cells15080671

**Published:** 2026-04-10

**Authors:** Raveen Badyal, Brandon Kohlen, Kevin J. Keen, James V. Dunne, Tillie-Louise Hackett

**Affiliations:** 1 Centre for Heart Lung Innovation, St. Paul’s Hospital, Vancouver, BC V6Z 1Y6, Canada; raveen.badyal@hli.ubc.ca (R.B.); brandon.kohlen@hli.ubc.ca (B.K.); kevin.keen@hli.ubc.ca (K.J.K.); james.dunne@vch.ca (J.V.D.); 2 Department of Anesthesiology, Pharmacology and Therapeutics, The University of British Columbia, Vancouver, BC V6T 1Z3, Canada; 3 Department of Mathematics and Statistics, The University of Northern British Columbia, Prince George, BC V2N 4Z9, Canada; 4 Department of Medicine, The University of British Columbia, Vancouver, BC V6T 1Z3, Canada

**Keywords:** transforming growth factor-beta isoforms, fibrosis, dermal fibroblasts, lung fibroblasts, extracellular matrix production, myofibroblast differentiation, collagen gel model, inflammation, proliferation

## Abstract

**Highlights:**

**What are the main findings?**

- TGF-β2 and TGF-β3 induce stronger profibrotic responses than TGF-β1 in both dermal and lung fibroblasts, including increased collagen-I and fibronectin synthesis and enhanced secretion of fibrotic cytokines Interleukin (IL)-6 and IL-11, suggesting potential relevance to skin and lung fibrosis in systemic sclerosis.
- The TGF-β isoforms exhibit tissue-specific effects, with TGF-β2 and TGF-β3 promoting collagen-I contraction in dermal fibroblasts and myofibroblast differentiation in lung fibroblasts, reflecting distinct functional roles of fibroblasts within different tissues.

**What are the implications of the main findings?**

- The role of TGF-β2 and TGF-β3 in tissue fibrosis are less well-characterized than TGF-β1; our findings demonstrate isoform and tissue-specific effects in dermal and lung fibrosis, providing a rationale for further investigation in the context of skin and lung disease in systemic sclerosis.
- As fibroblasts are the primary effector cells driving fibrosis, isoform-specific targeting of TGF-β2 and TGF-β3 may enable improved therapeutic approaches for skin and lung fibrosis in patients with systemic sclerosis.

**Abstract:**

Systemic sclerosis (SSc) is a multi-organ autoimmune disease characterized by fibrosis of the skin and internal organs. Interstitial lung disease (ILD) is a major complication and leading cause of mortality in SSc. Transforming growth factor-β (TGF-β) has been implicated as a central mediator of fibrosis; however, while TGF-β1 has been extensively studied, the roles of TGF-β2 and TGF-β3 remain incompletely defined. Here, we systematically compared the effects of TGF-β1, TGF-β2, and TGF-β3 in dermal and lung fibroblasts, evaluating extracellular matrix synthesis and contraction, cytokine secretion, proliferation, and myofibroblast differentiation. TGF-β2 and TGF-β3 induced greater profibrotic cytokine release of Interleukin (IL)-6 and IL-11 and increased collagen-I and fibronectin synthesis compared with TGF-β1 in dermal and lung fibroblasts (all *p* < 0.05). TGF-β2 and TGF-β3 stimulated greater collagen-I contraction in dermal fibroblasts (*p* < 0.05), but greater myofibroblast differentiation in lung fibroblasts (*p* < 0.05). The TGF-β isoforms did not affect proliferation. All TGF-β isoforms activated SMAD2/3 signalling; however, TGF-β2 and TGF-β3 reduced expression of TGF-β Receptor II and the inhibitory regulator, SMAD7. In summary, TGF-β2 and TGF-β3 have a more pronounced profibrotic effect than TGF-β1 on dermal and lung fibroblast functions, making them potential targets for treatment for skin and lung fibrosis in diseases such as SSc.

## 1. Introduction

Systemic sclerosis (SSc) is a rare autoimmune disorder with an estimated prevalence of 17 per 100,000 people worldwide, that predominantly affects women [1]. The main hallmark of SSc is skin fibrosis, which can be limited, affecting the face, below the knees, and below the elbows, or diffuse, affecting the whole body. In addition to skin fibrosis, patients with SSc present with organ fibrosis affecting the heart, kidneys, and digestive tract. Notably, 40% of SSc patients develop interstitial lung disease (ILD), which is associated with a 40% mortality rate within 10 years of diagnosis [2,3]. Currently, symptoms affecting the kidney, heart, and digestive tract are managed with use of angiotensin-converting enzyme (ACE) inhibitors, calcium channel blockers, and protein pump inhibitors. However, the mechanisms underlying skin and lung fibrosis in SSc remain poorly understood and there are no current cures. Thus, it is crucial to develop effective therapeutics for treating skin and lung fibrosis to improve quality of life and survival outcomes for patients with SSc.

Fibrosis is a maladaptive wound-healing response characterized by excessive extracellular matrix (ECM) deposition, persistent fibroblast activation, and progressive tissue stiffening that compromises organ function [4]. Fibroblasts, the most abundant cell type in the connective tissue of each organ, play a central role both in wound repair and fibrosis by producing and organizing ECM proteins and modulating the local immune response through the release of inflammatory mediators such as Interleukin (IL)-6 and IL-8 [4,5,6]. Fibroblast activity is tightly regulated by growth factors and cytokines released from the surrounding tissue microenvironment, including epithelial, stromal, and immune cells [7].

In terms of skin and lung fibrosis in SSc patients, the growth factor, transforming growth factor-beta (TGF-β), has been shown to be elevated in the blood and skin of SSc patients [8,9] and has been implicated in different fibrotic lung diseases through fibroblast activation [10,11]. TGF-β exists in three isoforms, TGF-β1, TGF-β2, and TGF-β3. While the effects of TGF-β1 have been extensively studied, the roles of TGF-β2 and TGF-β3, particularly in disease-relevant models of fibrosis, remain less well-defined. Previous studies have demonstrated that TGF-β1 activates fibroblasts in vitro, inducing ECM protein synthesis, including collagen-I and fibronectin, and promoting differentiation into contractile myofibroblasts with increased α-smooth muscle actin (α-SMA) expression [12,13]. Notably, gene expression profiles from skin biopsies of SSc patients resembles the gene signature of fibroblasts activated with TGF-β, specifically TGF-β3 [14]. While some groups have reported that TGF-β3 facilitates scarless wound healing, others report that TGF-β3 is indicative of a fibrotic disease signature [15,16]. These studies highlight the complexity of TGF-β isoform biology and the need to delineate their distinct roles in organ fibrosis in SSc. Although previous studies have examined the effect of one or two of the TGF-β isoforms on specific organ-derived fibroblasts [17,18], a direct comparison of all three TGF-β isoforms has not yet been conducted.

In this study, we systematically evaluated the effects of the three TGF-β isoforms, 1, 2, and 3, on human neonatal dermal and fetal lung fibroblast cell lines which are commercially available and non-transformed, enabling independent studies to replicate our results. Given that SSc manifests as a multi-organ fibrotic disease, we utilized both dermal and lung fibroblasts to model skin and lung-relevant responses to TGF-β in vitro, including ECM protein production and remodelling, fibroblast-to-myofibroblast differentiation, inflammatory and remodelling mediator release, proliferation, and TGF-β signalling pathway activation. Comparing these anatomically distinct fibroblast populations allows us to distinguish conserved versus tissue-specific effects of the TGF-β isoforms. Currently, most treatments targeting the TGF-β pathway inhibit the signalling of all TGF-β isoforms, and these inhibitors can lead to off-target and toxic side effects [19]. A deeper understanding of the TGF-β pathway could enable subsequent validation of TGF-β-isoform specific targets in adult SSc fibroblasts to safely ameliorate skin and lung fibrotic disease manifestations.

## 2. Materials and Methods

### 2.1. Cell Lines and Cell Culture Reagents

**Figure 1:**
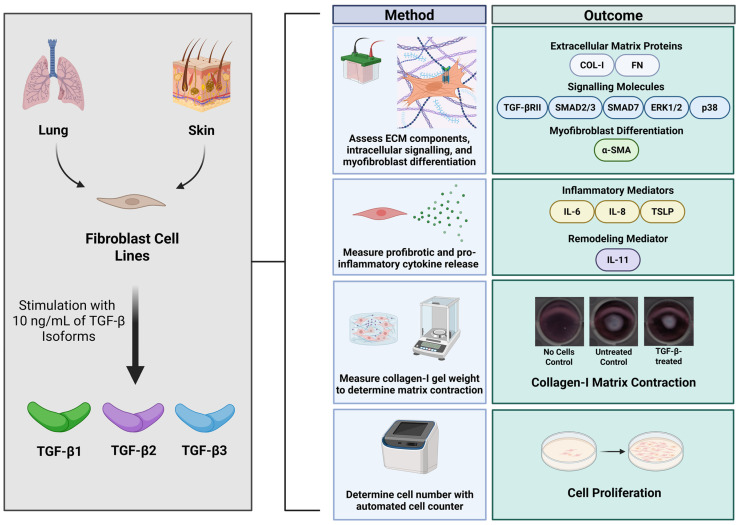
Study methodology. Fibroblast cell lines derived from neonatal skin and fetal lung tissue were stimulated with TGF-β isoforms 1, 2, and 3 at 10 ng/mL. Cells were phenotyped by Western blotting to assess extracellular matrix proteins, signalling molecules, and myofibroblast differentiation, ELISAs to measure inflammatory and remodelling cytokines, collagen-I matrix assays to assess fibroblast contraction, and a proliferation assay to assess the effect of the TGF-β isoforms on cell accumulation (Created in BioRender 201).

As shown in Figure 1, the study design used neonatal dermal fibroblasts (BJ, Cat: CRL-2522) and fetal lung fibroblasts (HFL1, Cat: CCL-153) obtained from the American Type Culture Collection (ATCC, Manassas, VA, USA), which are commonly used human, non-transformed dermal and lung fibroblast cell lines. Fibroblasts were seeded at 80,000 cells per well in 6-well plates and grown in 10% fetal bovine serum (FBS, Cat: 12483020, Thermo Fisher Scientific, Waltham, MA, USA) and 1% penicillin-streptomycin fungizone (PSF, Cat: SV3007901, Cytiva, Marlborough, MA, USA) in Dulbecco’s Modified Eagle Medium (DMEM, Thermo Fisher Scientific, Cat: 11965126) at 37 °C in 5% CO_2_, until 80% confluent. Fibroblasts were then serum-starved with DMEM containing 1% FBS for 16 h. Following review of prior publications and dose response experiments, dermal and lung fibroblasts were treated with 10 ng/mL of TGF-β1 (Cat: 78067, STEMCELL Technologies, Vancouver, BC, Canada), TGF-β2 (Cat: ab84070, Abcam, Waltham, MA, USA), or TGF-β3 (Thermo Fisher Scientific, Cat: 100-36E) for 30 min, 6 h, or 72 h, and compared to the control media (1% FBS and 1% PSF in DMEM). Experiments were conducted with fibroblasts at passages 1–5. The experiments were approved by the Providence Health Research Ethics Board (H13-02173) at the University of British Columbia.

### 2.2. Western Blot

Fibroblast protein was extracted using Cell Extraction Buffer (Thermo Fisher Scientific, Cat: FNN0011) supplemented with a phosphatase inhibitor (Cat: P5726-5ML, MilliporeSigma, Oakville, ON, Canada), a protease cocktail inhibitor (MilliporeSigma, Cat: P2714-1BTL), and phenylmethylsulfonyl fluoride (PMSF, MilliporeSigma, Cat: 10837091001). Total protein was quantified colorimetrically using a Pierce BCA Assay (Thermo Fisher Scientific, Cat: 22662) with the Spectramax iD3 plate reader (Molecular Devices, San Jose, CA, USA) and analyzed with SoftMax Pro 7. The cell lysates were prepared with 7.5 μg of protein and sample loading buffer (Cat: 928-40004, with β-mercaptoethanol, LI-COR Biotech, Lincoln, NE, USA), heated at 95 °C for 7 min, and run on 4–20% Mini-PROTEAN TGX Precast Protein Gels (Cat: 4561096, Bio-Rad Laboratories, Hercules, CA, USA). The gel was transferred onto a nitrocellulose membrane using a transfer buffer containing 20% methanol for 1 h at 100 volts. The blots were then stained for total protein using the Revert 700 Total Protein Stain Kit from LI-COR Biotech (Cat: 926-11016) according to the manufacturer’s protocol. After removing the Total Protein Stain, blots were blocked for 1 h in LI-COR Biotech Intercept Blocking Buffer (Cat: 927-60001) and probed with primary antibodies overnight at 4 °C. The primary antibodies included Collagen-I (1:1000, Abcam, Cat: ab138492), at 139 kDa, and Fibronectin (1:2000, Cat: sc-59826, Santa Cruz Biotechnology, Dallas, TX, USA), at 220 kDa, to assess ECM production. To investigate myofibroblast differentiation, α-smooth muscle actin (1:300, Thermo Fisher Scientific, Cat: PA5-85070) at 42 kDa was used. To assess TGF-β signalling, antibodies for the TGF-β Receptor II (TGF-βRII, 1:250, Cat: AF-241-NA, Bio-Techne, Minneapolis, MN, USA), Suppressor of Mothers Against Decapentaplegic (SMAD) 7 (1:500, Abcam, Cat: 216428), SMAD2/3 and phospho-SMAD2/3 (1:1000, Bio-Techne, Cat: AF3797 and 1:1000, Cell Signaling Technology, Cat: 8828), p38 and phospho-p38 (1:1000, Cat: 9212, Cell Signaling Technology, Danvers, MA, USA and 1:2000, Cell Signaling Technology, Cat: 9216S), and Extracellular Signal-Regulated Kinase (ERK) 1/2 and phospho-ERK1/2 (1:500, Thermo Fisher Scientific, Cat: 13-6200 and 1:1000, Thermo Fisher Scientific, Cat: MA5-38228) were used. The blots were incubated with their respective fluorescent secondary antibodies (1:5000, Cat: 926-32211, 926-68070, 926-68071, 926-32214, and 926-32212) from LI-COR for 1 h at room temperature, washed, and imaged using the LI-COR Odyssey CLx imager. Experiments were conducted using up to 11 biological replicates for both dermal and lung fibroblasts. Densitometry analysis was performed with the LICOR Image Studio 5.2 software.

### 2.3. Enzyme-Linked Immunosorbent Assay (ELISA)

Cell-free supernatants were used to determine the release of the cytokines Interleukin (IL)-6, IL-8, thymic stromal lymphopoietin (TSLP), and IL-11 using Duoset ELISAs (R&D, Minneapolis, MN, USA, Cat: DY206, DY208, DY218, DY1398, respectively) according to the manufacturer’s protocol. Experiments were conducted using 11 biological replicates for dermal fibroblasts and 10 biological replicates for lung fibroblasts. The assays were read at 450 nm with a reference wavelength of 570 nm using the Spectramax iD3 plate reader, and the SoftMax Pro 7 software was used to generate the standard curves.

### 2.4. Cell Proliferation

Following 72 h of TGF-β treatment, fibroblasts were trypsinized using 0.25% Trypsin-EDTA solution (Thermo Fisher Scientific, Cat: 25200072) and counted using Counting Chamber Slides (Thermo Fisher Scientific, Cat: C10283) and the Countess II Automated Cell Counter. The cell number of treated fibroblasts was normalized to the media control to account for differences among experiment replicates. Experiments were conducted using 6 biological replicates for both dermal and lung fibroblasts.

### 2.5. Lactate Dehydrogenase Assay

Cell-free supernatants were used to determine cell viability upon treatment with the TGF-β isoforms. Lactate dehydrogenase (LDH) release by fibroblasts was measured with an LDH assay kit from Abcam (Cat: ab65393) according to the manufacturer’s protocol. Experiments were conducted using 10 biological replicates for both dermal and lung fibroblasts. The Spectramax iD3 was used for colorimetric detection of LDH protein.

### 2.6. Collagen-I Gel Contraction Assay

Fibroblast contractility was measured using a collagen-I gel matrix contraction assay as previously described [20]. Briefly, 12-well tissue culture plates (Cat: 10062-894, Avantor, Radnor, PA, USA) were coated with DMEM containing 1% bovine serum albumin (BSA) for 2 h at room temperature. After removing the BSA solution from each well, 1 mL of rat tail collagen-I gel (Cat: CACB354236, Corning, Corning, NY, USA) at 0.4 mg/mL or 0.8 mg/mL was added to each well and incubated overnight at 37 °C. The collagen gels were detached from the walls of each well using a pipette tip, and the media was replaced before seeding the collagen-I gels with 40,000 cells. After 72 h, the gels were weighed using a fine balance to assess the ability of the fibroblasts to contract the gel, which leads to extrusion of the media and a reduction in gel weight. Experiments were conducted using 8 biological replicates for both dermal and lung fibroblasts. The data are presented as a percentage of collagen-I contraction.

### 2.7. Statistical Analysis

The Robust regression and Outlier removal method (ROUT) with a Q value of 10% was used to define and remove outliers. The data were tested for normality using a Shapiro–Wilk test and were found to be normally distributed. The data were analyzed for all assays using a one-way analysis of variance (ANOVA) with Tukey’s post hoc test between all treatment groups using GraphPad Prism 10.4.2 to assess the differences between the TGF-β isoforms. Data from all assays were also analyzed using Fisher’s Least Significant Difference (LSD) test, without correction for multiple comparisons to understand the individual TGF-β isoform effects compared to the media control; these results are provided in the Supplementary Materials. A *p*-value < 0.05 was considered significant.

## 3. Results

### 3.1. TGF-β2 and TGF-β3 Are Strong Inducers of Profibrotic IL-11 and IL-6 Release by Dermal and Lung Fibroblasts

**Figure 2:**
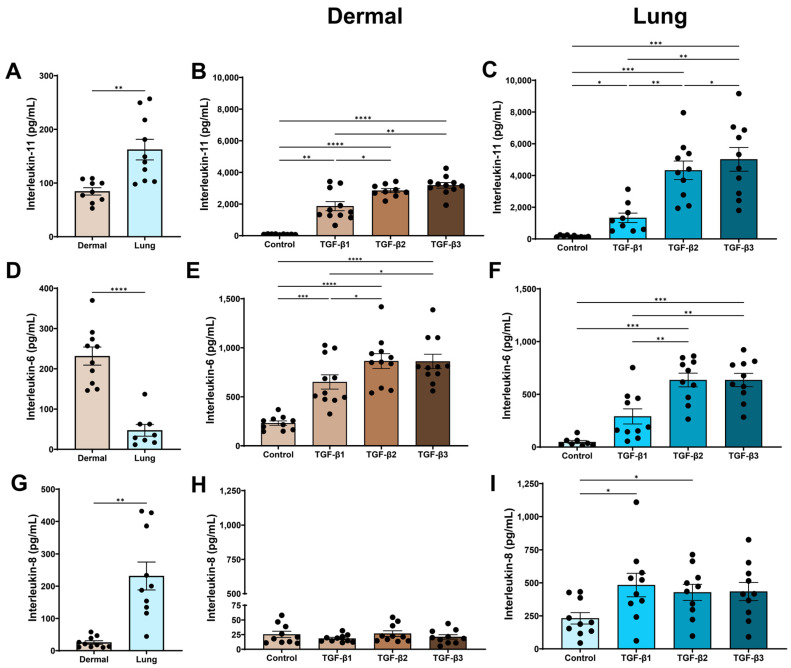
TGF-β2 and TGF-β3 are strong inducers of profibrotic IL-11 and IL-6 release by dermal and lung fibroblasts. Dermal and lung fibroblasts (*n* = 11 biological replicates for dermal fibroblasts and *n* = 10 for lung fibroblasts) were seeded in 6-well plates and stimulated with TGF-β isoforms 1, 2, and 3 for 72 h. Cell-free supernatant was collected and analyzed using ELISA to measure the concentration of (**A**–**C**) IL-11, (**D**–**F**) IL-6, and (**G**–**I**) IL-8. Data represent the mean and standard error of the mean (SEM). *t*-tests and ANOVA with Tukey’s post hoc test between groups were used to test for differences between groups. * (*p* < 0.05), ** (*p* < 0.01), *** (*p* < 0.001), **** (*p* < 0.0001).

IL-11, a member of the IL-6 family, is an important mediator of TGF-β-driven fibrosis in murine models [21], and promotes fibroblast-to-myofibroblast differentiation and collagen production [22]. At baseline, human lung fibroblasts released significantly more constitutive IL-11 than human dermal fibroblasts (*p* < 0.01, Figure 2A). All TGF-β isoforms increased IL-11 release in both dermal and lung fibroblasts (Figure 2B,C). However, TGF-β2 (*p* < 0.05, *p* < 0.01) and TGF-β3 (*p* < 0.01, *p* < 0.01) induced significantly greater IL-11 secretion than TGF-β1 in both dermal and lung fibroblasts, respectively (Figure 2B,C). In addition, lung fibroblasts stimulated with TGF-β3 released more IL-11 than lung fibroblasts stimulated with TGF-β2 (*p* < 0.05, Figure 2C).

IL-6 is an acute-phase inflammation cytokine that is upregulated in many autoimmune diseases and fibrotic diseases [23]. At baseline, dermal fibroblasts released significantly higher constitutive levels of IL-6 than lung fibroblasts (*p* < 0.0001, Figure 2D). All TGF-β isoforms increased IL-6 expression compared to the media control in dermal fibroblasts (Figure 2E). Both TGF-β2 (*p* < 0.05) and TGF-β3 (*p* < 0.05) were able to induce greater expression of IL-6 compared to TGF-β1 in dermal fibroblasts (Figure 2E). In lung fibroblasts, only TGF-β2 (*p* < 0.001) and TGF-β3 (*p* < 0.001) induced IL-6 expression, compared to the media control when using ANOVA with Tukey’s post hoc test (Figure 2F). However, using a less stringent Fisher’s LSD test, which does not account for multiple comparisons, TGF-β1 also induced a modest but significant increase in IL-6 release compared to the media control (Supplementary Figure S1A). In addition, TGF-β2 (*p* < 0.01) and TGF-β3 (*p* < 0.01) induced greater IL-6 expression compared to the TGF-β1-treated lung fibroblasts (Figure 2F).

IL-8 is a key neutrophil chemoattractant involved in tissue repair or injury responses [24,25,26]. At baseline, lung fibroblasts released significantly higher constitutive levels of IL-8 than dermal fibroblasts (*p* < 0.01, Figure 2G). TGF-β stimulation did not induce IL-8 release in dermal fibroblasts (Figure 2H). In contrast, lung fibroblasts demonstrated increased IL-8 expression following stimulation with TGF-β1 (*p* < 0.01) and TGF-β2 (*p* < 0.01), compared to the media control when compared using ANOVA with Tukey’s post hoc test (Figure 2I). When using Fisher’s LSD test, TGF-β3 also induced a significant increase in IL-8 release compared to the media control (Supplementary Figure S1B).

Thymic stromal lymphopoietin (TSLP), an alarmin involved in activation of the adaptive immune system and elevated in fibrosis [26], was not induced by any of the three TGF-β isoforms in either dermal or lung fibroblasts.

### 3.2. TGF-β2 and TGF-β3 Are Strong Inducers of Collagen-I and Fibronectin Production by Dermal and Lung Fibroblasts

**Figure 3:**
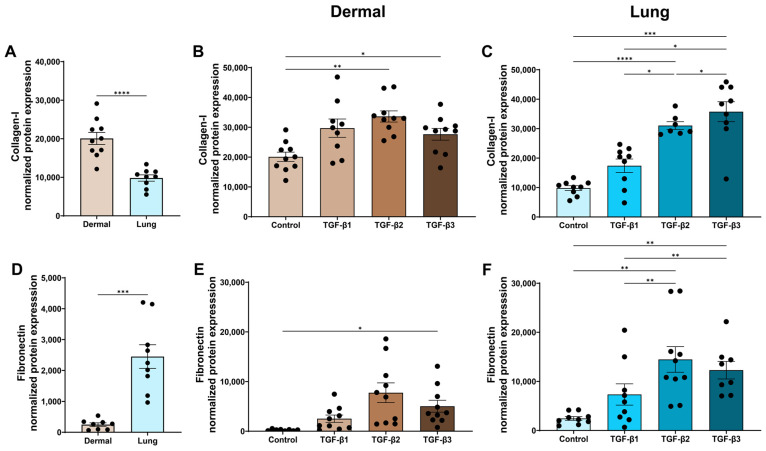
TGF-β2 and TGF-β3 are strong inducers of extracellular matrix production by dermal and lung fibroblasts. Dermal and lung fibroblasts (*n* = 10 biological replicates per cell line) were seeded in 6-well plates and stimulated with TGF-β isoforms 1, 2, and 3 for 72 h. Protein lysates were collected and analyzed using Western blotting to measure expression of (**A**–**C**) collagen-I and (**D**–**F**) fibronectin. Data represent the mean with SEM. *t*-tests and ANOVA with Tukey’s post hoc test between groups were used to test for differences between groups. * (*p* < 0.05), ** (*p* < 0.01), *** (*p* < 0.001), **** (*p* < 0.0001).

Collagens are an integral component of the ECM, providing structural support, tensile strength, and sites for cell adhesion and migration [27,28]. Collagen-I is the most abundant ECM collagen and is increased in skin and lung fibrosis [29,30,31]. At baseline, dermal fibroblasts produced more collagen-I protein than lung fibroblasts (*p* < 0.0001, Figure 3A). In dermal fibroblasts, only TGF-β2 and TGF-β3 induced a statistically significant increase in collagen-I protein expression when compared to the media control using ANOVA with Tukey’s post hoc test (Figure 3B). In lung fibroblasts, only TGF-β2 and TGF-β3 increased collagen-I expression compared to the media control (*p* < 0.0001 and *p* < 0.001) and TGF-β1-treated fibroblasts (*p* < 0.05 and *p* < 0.05, Figure 3C) using ANOVA with Tukey’s post hoc test. However, when using a less stringent Fisher’s LSD test that does not correct for multiple comparisons, all TGF-β isoforms induced collagen-I production in dermal and lung fibroblasts (Supplementary Figure S2A,B), and this reflects previous studies that had only tested one TGF-β isoform.

Fibronectin is a key ECM proteoglycan that binds multiple ECM proteins, signalling molecules, and cell adhesion receptors [32], and is elevated in fibrotic tissues [33,34]. At baseline, lung fibroblasts produced more fibronectin protein than dermal fibroblasts (*p* < 0.001, Figure 3D). In dermal fibroblasts, only the TGF-β3 isoform significantly increased production of fibronectin protein compared to the media control (Figure 3E). In lung fibroblasts, TGF-β2 and TGF-β3 significantly increased fibronectin protein compared with both the media control (*p* < 0.01 and *p* < 0.01) and TGF-β1 treatment (*p* < 0.01 and *p* < 0.01, Figure 3F). Again, using a non-stringent Fisher’s LSD test, all TGF-β isoforms induced fibronectin expression in dermal fibroblasts (Supplementary Figure S2C). In lung fibroblasts, TGF-β1 did increase fibronectin expression; however, this result was not significantly significant compared to the media control.

### 3.3. TGF-β2 and TGF-β3 Are Strong Inducers of Fibroblast-to-Myofibroblast Differentiation in Lung Fibroblasts

**Figure 4:**
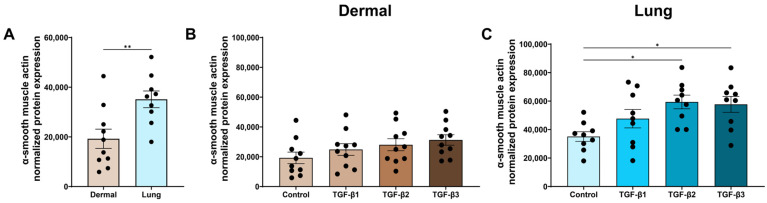
TGF-β2 and TGF-β3 are strong inducers of fibroblast-to-myofibroblast differentiation in dermal and lung fibroblasts. Dermal and lung fibroblasts (*n* = 10 biological replicates per cell line) were seeded in 6-well plates and stimulated with TGF-β isoforms 1, 2, and 3 for 72 h. Protein lysates were collected and analyzed using Western blotting to measure expression of α-smooth muscle actin of dermal and lung fibroblasts at baseline (**A**), dermal fibroblasts stimulated with the TGF-β isoforms (**B**), and lung fibroblasts stimulated with the TGF-β isoforms (**C**). Data represent the mean with SEM. *t*-tests and ANOVA with Tukey’s post hoc test between groups were used to test for differences between groups. * (*p* < 0.05), ** (*p* < 0.01).

During wound repair, fibroblasts can differentiate into contractile myofibroblasts to remodel the ECM [35]. TGF-β is a known inducer of fibroblast-to-myofibroblast differentiation, and this differentiation can be quantified using the formation of stress fibres, including α-SMA expression [36]. At baseline, lung fibroblasts expressed higher α-SMA protein levels than dermal fibroblasts (*p* < 0.01, Figure 4A). In dermal fibroblasts, none of the TGF-β isoforms induced α-SMA expression when corrected for multiple comparisons using ANOVA with Tukey’s post hoc test (Figure 4B). However, as shown in Supplementary Figure S2E, TGF-β1 (*p* < 0.05), TGF-β2 (*p* < 0.05), and TGF-β3 (*p* < 0.05) all induced α-SMA expression in dermal fibroblasts compared to the media control when using Fisher’s LSD test.

In lung fibroblasts, TGF-β2 and TGF-β3 increased α-SMA expression compared to the media control (*p* < 0.05 and *p* < 0.05, Figure 4C). However, as shown in Supplementary Figure S2F, TGF-β1 (*p* < 0.05), TGF-β2 (*p* < 0.01), and TGF-β3 (*p* < 0.05) induced α-SMA expression in lung fibroblasts compared to the media control when using Fisher’s LSD test.

### 3.4. TGF-β2 and TGF-β3 Are Strong Inducers of Fibroblast Contraction by Dermal Fibroblasts in a Fibrotic Environment

**Figure 5:**
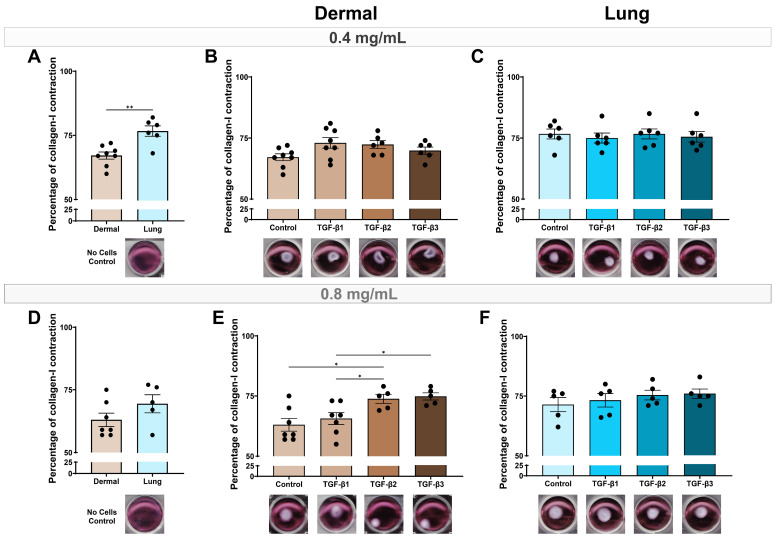
TGF-β2 and TGF-β3 are strong inducers of fibroblast contraction by dermal fibroblasts in a fibrotic environment. Dermal and lung fibroblasts (*n* = 5–8 biological replicates per cell line) were seeded on top of collagen-I gel matrices in 12-well plates and stimulated with TGF-β isoforms 1, 2, and 3 for 72 h. Collagen-I gel contraction was measured for gels at (**A**–**C**) 0.4 mg/mL and (**D**–**F**) 0.8 mg/mL. Data represent the mean with SEM. *t*-tests and ANOVA with Tukey’s post hoc test between groups were used to test for differences between groups. * (*p* < 0.05), ** (*p* < 0.01).

Fibroblast-mediated ECM contraction is essential for wound closure [37]. In a non-fibrotic ECM environment, represented by a 0.4 mg/mL collagen-I gel, lung fibroblasts were more contractile than dermal fibroblasts (*p* < 0.01, Figure 5A). Under these normal ECM conditions, none of the TGF-β isoforms increased contraction of dermal or lung fibroblasts (Figure 5B,C). In a fibrotic ECM, represented by a 0.8 mg/mL collagen-I gel matrix, baseline contraction did not differ between dermal and lung fibroblasts (Figure 5D). However, TGF-β2 (*p* < 0.05) and TGF-β3 (*p* < 0.05) significantly induced increased dermal fibroblast contraction compared to the media control and TGF-β1 (Figure 5E). Consistent with the non-fibrotic gel matrices, the TGF-β isoforms did not enhance the contraction of lung fibroblasts in the fibrotic gel matrices (Figure 5F).

### 3.5. The TGF-β Isoforms Do Not Influence Proliferation of Dermal and Lung Fibroblasts

**Figure 6:**
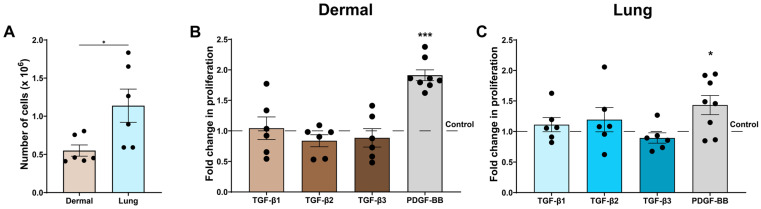
The TGF-β isoforms do not influence proliferation of dermal and lung fibroblasts. Dermal and lung fibroblasts (*n* = 6–8 biological replicates per cell line) were seeded in 6-well plates and stimulated with TGF-β isoforms 1, 2, and 3 and PDGF-BB for 72 h. Fibroblasts were detached and counted with an automated cell counter, normalized with the control treatment group, and reported as (**A**) number of cells or (**B**,**C**) fold change in proliferation. Data represent the mean with SEM. *t*-tests and ANOVA with Tukey’s post hoc test between groups were used to test for differences between groups. * (*p* < 0.05), *** (*p* < 0.001).

Fibroblast proliferation contributes to wound repair, providing additional cells to synthesize and contract the ECM. At baseline, lung fibroblasts proliferated more than dermal fibroblasts (Figure 6A). However, none of the TGF-β isoforms altered proliferation in either dermal or lung fibroblasts (Figure 6B,C). As a positive control, fibroblasts were treated with platelet-derived growth factor (PDGF)-isoform BB, a known stimulator of fibroblast proliferation [38,39,40], which induced a 1.9-fold increase in dermal and 1.4-fold increase in lung fibroblast proliferation after 72 h stimulation compared to the control media (Figure 6B,C). To exclude cytotoxic effects of TGF-β treatment, LDH release, a marker of cell death, was measured in the cell supernatant, and no increase in cell death was observed in the TGF-β isoform-treated fibroblasts compared to the control media (Supplementary Figure S3A,B).

### 3.6. The TGF-β Isoforms Are Strong Inducers of Canonical Signalling in Dermal and Lung Fibroblasts

**Figure 7:**
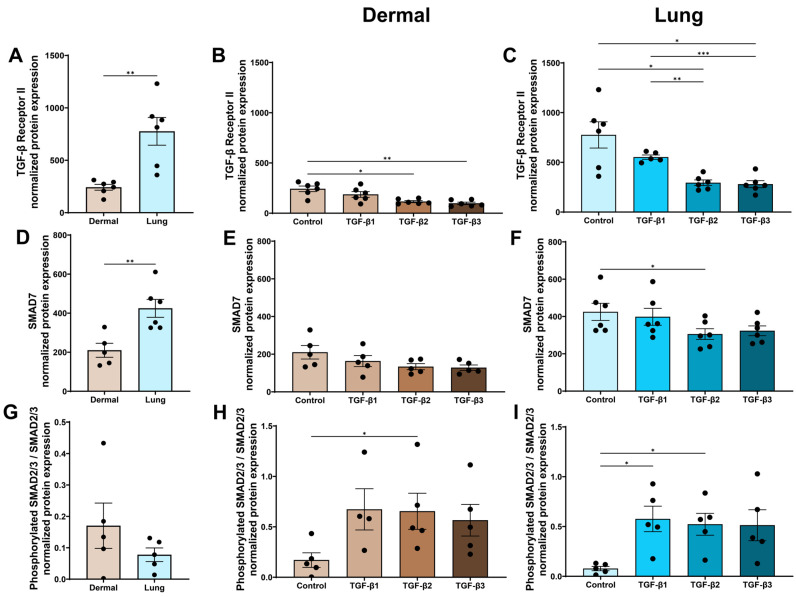
The TGF-β isoforms are strong inducers of canonical TGF-β signalling in dermal and lung fibroblasts. Dermal and lung fibroblasts (*n* = 6 biological replicates per cell line) were seeded in 6-well plates and stimulated with TGF-β isoforms 1, 2, and 3 for 30 min or 72 h. Protein lysates were collected and analyzed using Western blotting to measure expression of TGF-βRII (**A**–**C**), SMAD7 (**D**–**F**), and SMAD2/3 (**G**–**I**). Data represent the mean with SEM. *t*-tests and ANOVA with Tukey’s post hoc test between groups were used to test for differences between groups. * (*p* < 0.05), ** (*p* < 0.01), *** (*p* < 0.001).

TGF-β1 and TGF-β3 signal by binding first to TGF-β Receptor II (TGF-βRII), which then recruits TGF-β Receptor I (TGF-βRI), allowing for TGF-βRI to be phosphorylated, leading to signalling within the cell, whereas TGF-β2 also requires TGF-βRIII for efficient receptor engagement with TGF-βRII. When TGF-β initiates signalling, it also induces the downregulation of TGF-βRII as an autocrine feedback mechanism to switch off signalling. At baseline, lung fibroblasts express more TGF-βRII than dermal fibroblasts (*p* < 0.01, Figure 7A). In dermal fibroblasts, TGF-β2 and TGF-β3 reduced TGF-βRII expression compared to the media control (*p* < 0.05 and *p* < 0.01, Figure 7B). Similarly, in lung fibroblasts, TGF-β2 and TGF-β3 decreased TGF-βRII expression compared to control (*p* < 0.05 and *p* < 0.05) and TGF-β1 treatment (*p* < 0.01 and *p* < 0.001, Figure 7C).

Once TGF-βR1 is phosphorylated, signalling can occur through the canonical pathways involving SMAD2/3 or non-canonical pathways including p38, ERK1/2, PI3K, and Rho-like GTPase signalling. SMAD7 is the inhibitory regulator of the canonical SMAD2/3 pathway. At baseline, lung fibroblasts express increased SMAD7 compared to dermal fibroblasts (*p* < 0.01, Figure 7D). In dermal fibroblasts, none of the TGF-β isoforms changed SMAD7 expression when corrected for multiple comparisons using ANOVA with Tukey’s post hoc test (Figure 7E). However, as shown in Supplementary Figure S4C, TGF-β2 (*p* < 0.05) and TGF-β3 (*p* < 0.05) decreased SMAD7 expression in dermal fibroblasts compared to the media control when using Fisher’s LSD test. In lung fibroblasts, SMAD7 protein expression was decreased by the TGF-β2 treatment compared to the media control, using ANOVA and Tukey’s post hoc test (Figure 7F). However, as shown in Supplementary Figure S4D, TGF-β2 and TGF-β3 (*p* < 0.05) both decreased SMAD7 expression in lung fibroblasts when compared to the media control using Fisher’s LSD test.

At baseline, dermal fibroblasts expressed more phosphorylated SMAD2/3 than lung fibroblasts, but this result was not statistically significant (Figure 7G). In dermal fibroblasts, TGF-β2 increased phosphorylated SMAD2/3 compared to the media control using ANOVA with Tukey’s post hoc test (*p* < 0.05, Figure 7H). In lung fibroblasts, TGF-β1 and TGF-β2 increased phosphorylated SMAD2/3 using an ANOVA with Tukey’s post hoc test (*p* < 0.05, Figure 7I). However, all TGF-β isoforms activated canonical SMAD2/3 signalling in dermal and lung fibroblasts (Supplementary Figure S4E,F) compared to the media control when using Fisher’s LSD test.

### 3.7. The TGF-β Isoforms May Have Differential Effects on Non-Canonical TGF-β Signalling in Dermal and Lung Fibroblasts

**Figure 8:**
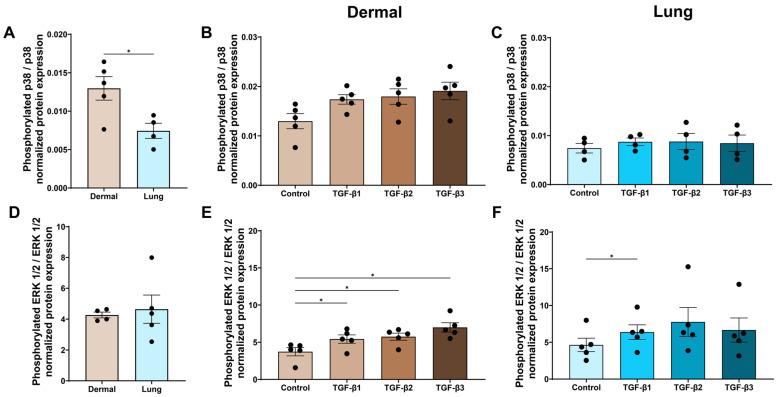
The TGF-β isoforms may have differential effects on non-canonical TGF-β signalling in dermal and lung fibroblasts. Dermal and lung fibroblasts (*n* = 5 biological replicates per cell line) were seeded in 6-well plates and stimulated with TGF-β isoforms 1, 2, and 3 for 6 h. Protein lysates were collected and analyzed using Western blotting to measure expression of p38 (**A**–**C**) and ERK1/2 (**D**–**F**). Data represent the mean with SEM. *t*-tests and ANOVA with Tukey’s post hoc test between groups were used to test for differences between groups. * (*p* < 0.05).

To investigate the effects of the TGF-β isoforms on non-canonical TGF-β signalling, we assessed the expression of phosphorylated p38 and ERK1/2. At baseline, dermal fibroblasts expressed more phosphorylated p38 compared to lung fibroblasts (*p* < 0.05, Figure 8A). In dermal fibroblasts, signalling of p38 was activated with TGF-β stimulation, but this result was not statistically significant when using an ANOVA with Tukey’s post hoc test (Figure 8B). When using Fisher’s LSD test, not correcting for multiple comparisons, only dermal fibroblasts stimulated with TGF-β1 increased the expression of phosphorylated p38 (*p* < 0.05, Supplementary Figure S5A). In lung fibroblasts, there was a small increase in phosphorylated p38 when comparing the TGF-β treatment groups to the media control, but that increase was not statistically significant (Figure 8C). Non-canonical ERK1/2 signalling was significantly increased by all three TGF-β isoforms in dermal fibroblasts (Figure 8E). In lung fibroblasts, all TGF-β isoforms increased phosphorylation of ERK1/2, but TGF-β1 significantly increased ERK1/2 signalling compared to the control when using an ANOVA with Tukey’s post hoc test to correct for multiple comparisons (*p* < 0.05, Figure 8F). When using Fisher’s LSD test, TGF-β2 also increased phosphorylated ERK1/2 expression compared to control media and TGF-β3 treatment in lung fibroblasts (*p* < 0.05 and *p* < 0.05, Supplementary Figure S5D).

## 4. Discussion

In this study, using stringent multiple comparisons testing, we demonstrate that TGF-β2 and TGF-β3 exert more pronounced profibrotic effects than TGF-β1 in both dermal and lung fibroblasts. Specifically, TGF-β2 and TGF-β3 induced greater ECM production, including collagen-I and fibronectin, and enhanced secretion of fibrotic cytokines, IL-6 and IL-11, compared to TGF-β1 in both dermal and lung fibroblasts. However, TGF-β2 and TGF-β3 demonstrated tissue-specific isoform effects, including greater collagen-I contraction in dermal fibroblasts and enhanced myofibroblast differentiation in lung fibroblasts. These findings highlight that, while TGF-β1 is the most studied isoform in fibrosis research, TGF-β2 and TGF-β3 may play significant roles in driving fibroblast dysfunction in skin and lung fibrosis in diseases such as systemic sclerosis. Differential regulation of SMAD2/3 signalling, via SMAD7 inhibition, and non-canonical ERK1/2 signalling were associated with these TGF-β isoform-specific effects in dermal and lung fibroblasts. However, further work investigating TGF-β signalling in adult-derived SSc fibroblasts will be necessary to confirm the reported TGF-β isoform-specific effects. Additionally, tissue fibrosis is a feature of various skin pathologies, including hypertrophic scars, keloids, and morphea, as well as lung diseases such as idiopathic pulmonary fibrosis and pneumoconiosis, suggesting that the findings from this study may have broader relevance beyond systemic sclerosis.

A hallmark feature of fibrosis is excessive ECM deposition, leading to increased tissue stiffness and impaired loss of organ function. At baseline, dermal fibroblasts produced more collagen-I, whereas lung fibroblasts produced more fibronectin. These results highlight that while fibroblasts and their synthesized ECM are throughout the body, there are organ-specific fibroblast phenotypes and ECM compositions [41,42]. While TGF-β1 is well established as a key inducer of ECM proteins [43], the effects of TGF-β2 and TGF-β3 have been less characterized. This study highlights that with stringent testing for multiple comparisons, TGF-β2 and TGF-β3 were dominant drivers of ECM synthesis in dermal and lung fibroblasts compared to TGF-β1. This finding is supported by transcriptomic studies showing elevated TGF-β3 expression in skin biopsies from SSc patients [14] and increased TGF-β2 expression in fibrotic lung tissue [16]. These differences likely reflect organ-specific fibroblast phenotypes and microenvironmental cues that modulate TGF-β responsiveness [44]. Previous studies have shown that pan-inhibition of the TGF-β isoforms was effective at reducing skin fibrosis in patients with SSc; however, patients experienced adverse effects such as bleeding disorders and anemia [19]. Our findings therefore support the rationale for isoform-selective targeting of TGF-β to reduce side effects but also focus on the potent TGF-β2 and TGF-β3 isoforms.

Fibroblast activation and differentiation into a contractile myofibroblast with an α-SMA-positive cytoskeleton is a key function in wound repair to provide contractile force and increased ECM synthesis for wound closure. The number and persistence of myofibroblasts have been shown to increase due to proliferation and increased resistance to apoptosis in the skin and the lungs of patients with SSc [45]. Within the literature, a 10 ng/mL dose of TGF-β1 has been shown to induce fibroblast-to-myofibroblast differentiation in primary fibroblasts isolated from the skin and the lung [41,46]. In this study comparing the three isoforms with statistical testing for multiple comparisons, TGF-β2 and TGF-β3 were the strongest inducers of α-SMA in lung fibroblasts compared to the control media. While TGF-β1 treatment did not survive robust multiple-comparison testing, when using Fisher’s LSD test without multiple comparisons testing as shown in the Supplementary Materials, TGF-β1 was able to induce α-SMA expression in lung fibroblasts as previously reported in other studies. Thus, these findings highlight the importance of testing all isoforms together to decern statistically significant and functionally relevant differences in the TGF-β isoforms on fibroblast functions.

For the collagen-I matrix contraction assay, lung fibroblasts were more contractile than dermal fibroblasts at baseline, which may reflect the elevated α-SMA expression in lung fibroblasts at baseline compared to dermal fibroblasts. This may also reflect why dermal fibroblasts were more responsive in contraction to the TGF-β isoforms than lung fibroblasts, particularly under fibrotic conditions, where TGF-β2 and TGF-β3 significantly enhanced matrix contraction. Previous studies show that fibroblasts from active wounds are more contractile in a collagen gel than healthy fibroblasts, highlighting the ability for activated dermal fibroblasts to be more contractile at different stages of wound repair [47,48]. Further, concurrent with this study, dermal fibroblasts contract collagen matrices differently when compared to lung fibroblasts [49]. The findings in this study suggest that the TGF-β isoforms may have stage-dependent roles in wound repair, with TGF-β2 and TGF-β3 contributing more to established fibrosis rather than early activation. However, this interpretation requires validation in fibroblasts isolated from fibrotic skin and lungs, such as in SSc.

Beyond ECM synthesis and contraction, fibroblasts are increasingly recognized as immunomodulatory effectors that influence tissue inflammation, repair, and fibrosis through cytokine and chemokine release. A number of epithelial-derived proinflammatory mediators and damage-associated molecular patterns (DAMPs) such as IL-1α, IL-1β [5,20], tumour necrosis factor-α, and IL-6 have been shown to cause fibroblast activation and subsequent cytokine release in both dermal and lung fibroblasts [50,51]. In this study, we found that all three TGF-β isoforms stimulate IL-6 secretion, with TGF-β2 and TGF-β3 eliciting the strongest response in dermal and lung fibroblasts. IL-6 is a central mediator, linking inflammation with fibrosis. Elevated IL-6 expression has been reported in both skin and lung fibrosis [23,52]. Mechanistically, IL-6 has been shown to promote proliferation, fibroblast-to-myofibroblast differentiation, and collagen synthesis via STAT3-dependent signalling in dermal fibroblasts [53]. In support of our findings, primary human adult lung fibroblasts stimulated with TGF-β have been shown to have increased activation and increased secretion of IL-6 [54,55]. In addition, IL-6 has been found to be increased in the bleomycin-induced pulmonary fibrosis murine model, and inhibition of IL-6 downregulated markers of TGF-β-induced fibrosis, such as collagen-I and α-SMA [56]. Further, the clinical efficacy of IL-6 blockade with tocilizumab in SSc-ILD demonstrates that targeting the IL-6 pathway can reduce inflammation and slow pulmonary decline, although responses are variable in patients, suggesting the mediators downstream of TGF-β signalling are involved in fibrosis [57].

Among other fibrotic mediators, IL-11 is upregulated in fibrotic conditions, and has recently emerged as a critical downstream effector of TGF-β signalling [21,58]. TGF-β is known to upregulate the transcription and secretion of IL-11 by primary human adult dermal and lung fibroblasts [59,60,61]. In this study, TGF-β2 and TGF-β3 robustly induced IL-11 protein secretion, up to four-fold higher than IL-6. Prior studies have demonstrated that fibroblasts derived from patients with systemic sclerosis express higher levels of the IL-11 receptor relative to the IL-6 receptor [62], which may explain the greater production of IL-11 than IL-6 observed in our study. IL-11 has been shown to drive fibrosis in the bleomycin-induced pulmonary fibrosis murine model, where its inhibition attenuated increased α-SMA fibroblast expression, collagen deposition, and tissue stiffness [21]. IL-11 acts through ERK-dependent pathways to promote fibroblast activation, ECM synthesis, and has been implicated in SSc fibroblast gene signatures [63,64]. Our findings suggest that TGF-β2 and TGF-β3 may mediate their strong profibrotic effects, in part, through IL-11 induction. Elevated IL-11 expression in serum and dermal fibroblasts from patients with SSc [65,66] further supports this axis as a promising therapeutic target.

Notably, the TGF-β isoforms did not broadly activate all inflammatory cytokines in fibroblasts. Dermal fibroblasts secreted minimal IL-8, and TSLP, and lung fibroblasts released only low levels of IL-8 when stimulated with the TGF-β isoforms, whereas previous studies have shown that dermal fibroblasts can release IL-8 in response to stimuli such as tumour necrosis factor-α and IL-1β [20,67]. This selective cytokine induction indicates that TGF-β2 and TGF-β3 preferentially engage profibrotic rather than proinflammatory programmes, reinforcing their role in driving fibrosis. In addition, responses observed in neonatal and fetal fibroblasts may differ from those of primary adult fibroblasts from patients with SSc. Furthermore, the interactions of immune cells with fibroblasts in vivo may alter the profile of the cytokines released.

During wound repair and fibrosis, an increase in the proliferation of fibroblasts is thought to accelerate ECM synthesis and remodelling [68]. Previous studies have reported mixed results on the effect of TGF-β on fibroblast proliferation [69,70,71,72]. Specifically, TGF-β1 had no effect on proliferation of healthy fetal lung fibroblasts or primary adult dermal fibroblasts [70,71,72]. Other studies have reported that TGF-β1 increased the proliferation of normal adult dermal fibroblasts and a neonatal dermal fibroblast cell line [69,70]. Thus, the lack of effect of TGF-β on proliferation in the current study with neonatal dermal and fetal lung fibroblasts, highlights the importance of future experiments with dermal and lung fibroblasts from patients with SSc to determine disease-specific effects of TGF-β on fibroblast proliferation. As stimulation with the positive control, PDGF-BB, did induce proliferation in the dermal and lung fibroblasts, these data demonstrate that the fibroblasts used in our study can be responsive to growth factors that influence proliferation in the cell culture conditions used.

To investigate why TGF-β2 and TGF-β3 exert dominant profibrotic effects on dermal and lung fibroblasts, we examined TGF-β receptor expression. All TGF-β isoforms, but particularly TGF-β2- and TGF-β3-induced TGF-βRII downregulation when correcting for multiple comparisons, indicate a robust autocrine signalling feedback response [73]. The three TGF-β isoforms signal through the same receptors, but there is limited literature addressing the differential effects of the TGF-β isoforms on canonical and non-canonical TGF-β signalling. Previous studies have shown that TGF-β and SMAD2/3 are upregulated in the skin tissue of patients with systemic sclerosis [74] and in the lung tissues of the murine bleomycin lung fibrosis model [75]. Canonical SMAD2/3 signalling was activated to a similar level by all TGF-β isoforms. SMAD7, the inhibitor of SMAD2/3 phosphorylation/complex formation, was significantly decreased by TGF-β2 and TGF-β3 compared to the media control and TGF-β1 treatment using non-stringent testing, and, thus, could enable the enhanced signalling responses seen in response to TGF-β2 and TGF-β3 compared to TGF-β1.

In dermal fibroblasts, non-canonical signalling through phosphorylation of p38 was elevated at baseline, but only ERK1/2 signalling was increased with all TGF-β isoforms. In lung fibroblasts, p38 was not increased with TGF-β stimulation. However, ERK1/2 was increased significantly by TGF-β1 and TGF-β2 compared to the media control in lung fibroblasts. As IL-11 secretion is known to be increased with the upregulation of the ERK1/2 pathway in dermal and lung fibroblasts [21,62], activation of ERK1/2 signalling in this study may contribute to the elevated release of IL-11 by dermal and lung fibroblasts. Signalling of p38 is known to be upregulated upon TGF-β stimulation [76,77], but our study found only a minimal increase in p38 in dermal fibroblasts. Following review of the literature, we found no studies assessing the differential response of canonical and non-canonical signalling to TGF-β stimulation in dermal or lung fibroblasts. Further studies will be required to determine if the differential effects of the TGF-β isoforms on fibroblast functions also occur in adult dermal and lung fibroblasts and if these are due to differential downstream canonical and non-canonical signalling.

Across all functional assays, dermal and lung fibroblasts exhibited distinct baseline phenotypes, underscoring intrinsic, tissue-specific programming that likely shapes fibrotic responses. At baseline, dermal fibroblasts produced higher levels of collagen-I, consistent with their primary role in maintaining tensile strength and structural integrity of the skin and were more contractile in a 0.8 mg/mL collagen-I gel, which is representative of the collagen concentration within the skin [78]. In contrast, lung fibroblasts preferentially produced fibronectin, a key provisional matrix protein, which is known to be more dynamically regulated in pulmonary tissue and exhibits higher turnover in lung fibroblasts compared to dermal fibroblasts [79]. Lung fibroblasts also demonstrated higher baseline IL-11 and α-SMA expression, consistent with literature showing that pulmonary fibroblasts more readily adopt or maintain a myofibroblast-like phenotype, particularly in the context of tissue repair and fibrotic remodelling [80]. These findings align with the broader concept of fibroblast heterogeneity, whereby fibroblast phenotype and function are shaped by anatomical location, developmental origin, and local microenvironmental cues, resulting in distinct transcriptional and functional profiles across organs [41,81]. However, such baseline differences did not influence how each fibroblast population responded to the TGF-β-isoforms, with dermal and lung fibroblasts, both responding to TGF-β2 and TGF-β3 with enhanced ECM synthesis and myofibroblast differentiation. While these findings will require further validation in adult fibroblasts, the neonatal dermal and fetal lung fibroblast cell lines indicate that there is not a single dominant TGF-β isoform for therapeutic targeting in skin or lung fibrosis, but that modulation of TGF-β2 and TGF-β3 may be required to effectively attenuate fibrotic fibroblast responses.

Limitations of this study include the use of human fetal lung (HFL1) and neonatal dermal (BJ) cell lines that may not fully recapitulate adult or disease-specific fibroblast phenotypes. However, these cell lines are not virus-transformed and provide a robust, reproducible model that can be replicated by researchers, as they are commercially available. Future research would benefit from the use of adult primary fibroblasts from healthy individuals and patients with SSc to better understand the role of the TGF-β isoforms on fibroblasts in healthy wound repair and fibrosis in the skin and lung. Second, the in vitro monoculture used in this study does not recapitulate the involvement of immune cells and other structural cells such as the lung epithelium or epidermis which interact with fibroblasts in vivo. Further work using complex models, such as fibroblasts with immune cell or epithelial cell cocultures, will be helpful to understand the role of each TGF-β isoform on cell–cell interactions in wound repair and fibrosis. Third, we focused on prominent ECM proteins in fibrosis, collagen-I and fibronectin; however, the expression of other ECM proteins may be differentially affected by the TGF-β isoforms. Fourth, the 3D collagen gel matrices used in this study consisted only of collagen-I. While collagen-I is the most abundant ECM protein in the body, we did not assess how TGF-β may affect the contraction of fibrillar and non-fibrillar ECM proteins such as collagen III or IV in the skin and lung. Lastly, we only assessed the p38 and ERK1/2 non-canonical signalling pathways and not PI3K and Rho-like GTPase, the additional non-canonical pathways.

## 5. Conclusions

While TGF-β1-driven mechanisms of fibrosis have been the focus of the majority of studies, this study demonstrates that TGF-β2 and TGF-β3 can drive stronger profibrotic responses in dermal and lung fibroblasts than TGF-β1 when accounting for multiple-comparison testing. These results support a shift from a TGF-β1-centric focus in fibrosis to a better understanding of isoform-specific TGF-β mechanisms in fibrosis of the skin and lung. Further validation of TGF-β2 and TGF-β3 signalling in adult-derived SSc fibroblasts will be crucial to determine if precision therapies for TGF-β signalling in patients with SSc can ameliorate skin and lung fibrosis with reduced side effects.

## Data Availability

The raw data supporting the conclusions of this article will be made available by the authors on request.

## Supplementary Materials

The following supporting information can be downloaded at: https://www.mdpi.com/article/10.3390/cells15080671/s1, Figure S1: All TGF-β isoforms induce Il-6 and IL-8 release by lung fibroblasts compared to the media control when not adjusting for multiple comparisons; Figure S2: All TGF-β isoforms induce dermal and lung fibroblast production of collagen-1, fibronectin and α-smooth muscle actin when not adjusting for multiple comparisons; Figure S3: The TGF-β isoform treatments were not cytotoxic to dermal or lung fibroblasts; Figure S4: TGF-β2 and TGF-β3 downregulate TGF-βRII and SMAD7, all TGF-β isoforms induce canonical SMAD2/3 signalling when not adjusting for multiple comparisons; Figure S5: Non-canonical TGF-β pathways, p38 and ERK1/2, are induced similarly by the TGF-β isoforms in dermal and lung fibroblasts when not adjusting for multiple comparisons.

## Abbreviations

The following abbreviations are used in this manuscript:

TGF-β	Transforming Growth Factor-β
IL	Interleukin
ECM	Extracellular Matrix
ILD	Interstitial Lung Disease
SSc	Systemic Sclerosis
α-SMA	α-Smooth Muscle Actin
FBS	Fetal Bovine Serum
PSF	Penicillin Streptomycin Fungizone
DMEM	Dulbecco’s Modified Eagle Medium
TGF-βRII	TGF-β Receptor II
SMAD	Suppressor of Mothers Against Decapentaplegic
ERK	Extracellular Signal-Regulated Kinase
TSLP	Thymic Stromal Lymphopoietin
LDH	Lactate Dehydrogenase
BSA	Bovine Serum Albumin
ANOVA	Analysis of Variance
LSD	Least Significant Difference
SEM	Standard Error of the Mean
3D	Three-Dimensional
