# Supplementary material for: Systematic Comparison of the TGF-β Isoforms in Normal Dermal and Lung Fibroblasts Identifies TGF-β2 and TGF-β3 as Priority Targets in Tissue Fibrosis
Source: Cells. 2026 Apr 10;15(8):671. doi: 10.3390/cells15080671 (PMC13114339; doi:10.3390/cells15080671)
Supplement: Supplementary file 1 [file cells-15-00671-s001.zip › cells-4200101-supplementary.pdf]

Supplementary Materials

Systematic Comparison of the TGF-β Isoforms in Normal Dermal and Lung Fibroblasts Identifies TGF-β2 and TGF-β3 as Priority Targets in Tissue Fibrosis

Supplementary Results

Supplementary Figure S1. All TGF-β isoforms induce IL-6 and IL-8 release by lung fibroblasts compared to the media control when not adjusting for multiple comparisons

In lung fibroblasts, all TGF-β isoforms increased the release of Interleukin (IL)-6 and IL-8 cytokines compared to the media control, when using the less stringent Fisher's Least Significant Difference (LSD) test, which does not correct for multiple comparisons (Supplementary Figure S1A,B).

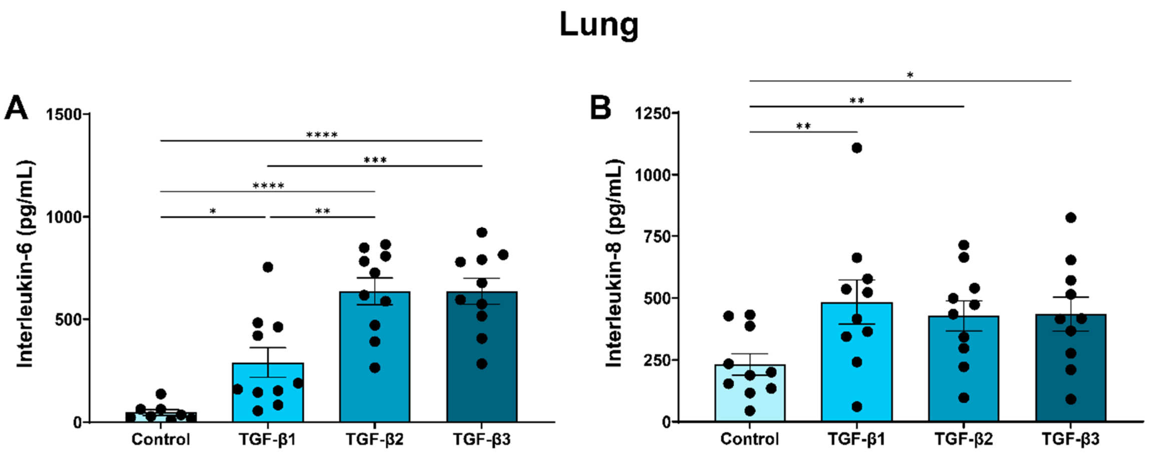

Supplementary Figure S1. All TGF-β isoforms induce Il-6 and IL-8 release by lung fibroblasts compared to the media control when not adjusting for multiple comparisons. Dermal and lung fibroblasts (n = 10 biological replicates) were seeded in 6-well plates and stimulated with TGF-β isoforms 1, 2, and 3 for 72 h. Cell-free supernatant was collected and analyzed using ELISA to measure the concentration of (**A**) IL-6 and (**B**) IL-8. Data represent the mean and standard error of the mean (SEM). The Fisher's LSD test was used to test differences between treatment groups without correcting for multiple comparisons. *(p<0.05), **(p<0.01), ***(p<0.001), ****(p<0.0001).

Supplementary Figure S2: All TGF-β isoforms induce dermal and lung fibroblast production of collagen-1, fibronectin, and α-smooth muscle actin when not adjusting for multiple comparisons

All TGF-β isoforms induced dermal and lung fibroblast production of collagen-1, fibronectin, and α-smooth muscle actin, when performing the Fisher's LSD test, which does not correct for multiple comparisons (Supplementary Figure S2). TGF-β1 increased fibronectin production by lung fibroblasts compared to the media control, but this difference was not statistically significant (Supplementary Figure S2D).

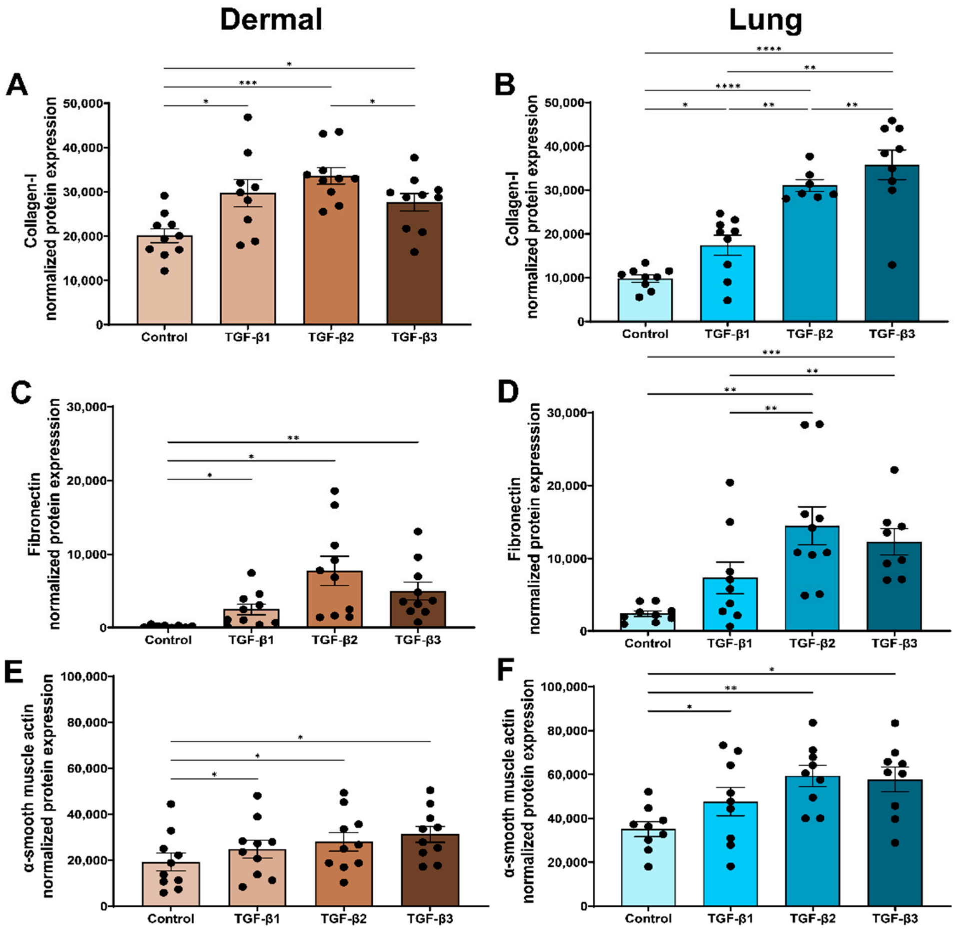

Supplementary Figure S2. All TGF-β isoforms induce dermal and lung fibroblast production of collagen-1, fibronectin, and α-smooth muscle actin when not adjusting for multiple comparisons. Dermal and lung fibroblasts (n = 10 biological replicates per cell line) were seeded in 6-well plates and stimulated with TGF-β isoforms 1, 2, and 3 for 72 h. Protein lysates were collected and analyzed using Western blotting to measure expression of (**A–B**) collagen-I, (**C–D**) fibronectin, and (**E–F**) α-smooth muscle actin. Data represent the mean with SEM. The Fisher's LSD test was used to test differences between treatment groups without correcting for multiple comparisons. *($p<0.05$), **($p<0.01$), ***($p<0.001$), ****($p<0.0001$).

Supplementary Figure S3: The TGF-β isoform treatments were not cytotoxic to dermal or lung fibroblasts

To measure if the TGF-β treatments had cytotoxic effects on the dermal and lung fibroblasts, a lactate dehydrogenase (LDH) assay was performed. The TGF-β treatments did not have a cytotoxic effect on the dermal or lung fibroblasts as the LDH protein released by the treated fibroblasts is not increased compared to the media control and remains low when compared to the positive control used for this assay (Supplementary Figure S3A,B). An aliquot of 500,000 dermal or lung fibroblasts were lysed and used as the positive control for this assay.

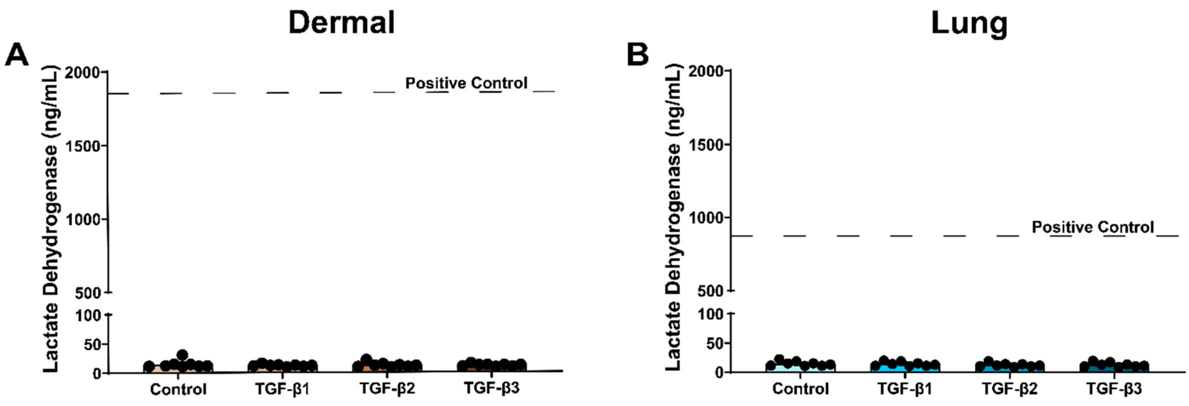

Supplementary Figure S3. The TGF-β isoform treatments were not cytotoxic to dermal (**A**) or lung (**B**) fibroblasts. Dermal and lung fibroblasts (n = 10 biological replicates per cell line) were seeded in 6-well plates and stimulated with TGF-β isoforms 1, 2, and 3 for 72 h. Cell-free supernatant was collected and analyzed using a lactate dehydrogenase (LDH) cytotoxicity assay to measure the concentration of LDH protein released by the fibroblasts. Data represent the mean with SEM. ANOVA with a Tukey's post-hoc test between groups was used to test for differences between groups. The Positive Control represents the amount of LDH protein released upon lysis of 500,000 dermal or lung fibroblasts.

Supplementary Figure S4: TGF-β2 and TGF-β3 downregulate TGF-βRII and SMAD7, and all TGF-β isoforms induce canonical SMAD2/3 signalling when not adjusting for multiple comparisons

In dermal and lung fibroblasts, TGF-βRII was downregulated in fibroblasts treated with TGF-β2 and TGF-β3 compared to the media control and fibroblasts treated with TGF-β1, when using the Fisher's LSD test (Supplementary Figure S4A,B). In dermal and lung fibroblasts, SMAD7 was downregulated by TGF-β2 and TGF-β3 compared to the media control using the Fisher's LSD test (Supplementary Figure S4C,D). In addition, TGF-β3 downregulated SMAD7 compared to TGF-β1 in the lung fibroblasts (Supplementary Figure S4D). All TGF-β isoforms induced canonical signalling through the SMAD2/3 pathway in dermal and lung fibroblasts, when using a Fisher's LSD test (Supplementary Figure S4E,F).

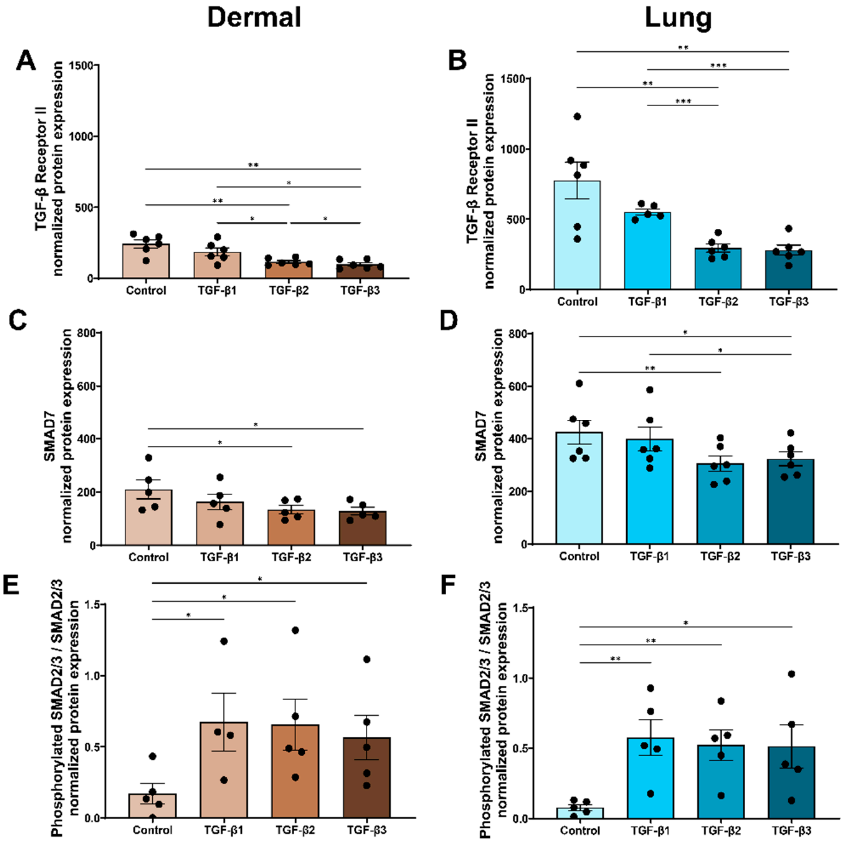

Supplementary Figure S4. TGF-β2 and TGF-β3 downregulate TGF-βRII and SMAD7, and all TGF-β isoforms induce canonical SMAD2/3 signalling when not adjusting for multiple comparisons. Dermal and lung fibroblasts (n = 6 biological replicates per cell line) were seeded in 6-well plates and stimulated with TGF-β isoforms 1, 2, and 3 for 30 min or 72 h. Protein lysates were collected and analyzed using Western blotting to measure expression of TGF-βRII (**A–B**), SMAD7 (**C–D**), and SMAD2/3 (**E–F**). Data represent the mean with SEM. The Fisher's LSD test was used to test differences between treatment groups without correcting for multiple comparisons. *($p<0.05$), **($p<0.01$), ***($p<0.001$).

Supplementary Figure S5: Non-canonical TGF-β pathways, p38 and ERK1/2, are induced similarly by the TGF-β isoforms in dermal and lung fibroblasts when not adjusting for multiple comparisons

In dermal fibroblasts, all TGF-β isoforms increased non-canonical p38 signalling; however, TGF-β1 increased phosphorylated p38 significantly compared to the media control, using a Fisher's LSD test (Supplementary Figure S5A). Non-canonical p38 signalling was not induced by the TGF-β isoforms in lung fibroblasts (Supplementary Figure S5B). In dermal fibroblasts, all TGF-β isoforms increased phosphorylated ERK1/2 expression significantly, when using a Fisher's LSD test (Supplementary Figure S5C). In lung fibroblasts, TGF-β1 and TGF-β2 increased phosphorylated ERK1/2 expression compared to the media control, when using the Fisher's LSD test (Supplementary Figure S5D). In addition, TGF-β2 increased ERK1/2 expression compared to the lung fibroblasts treated with TGF-β3 (Supplementary Figure S5D).

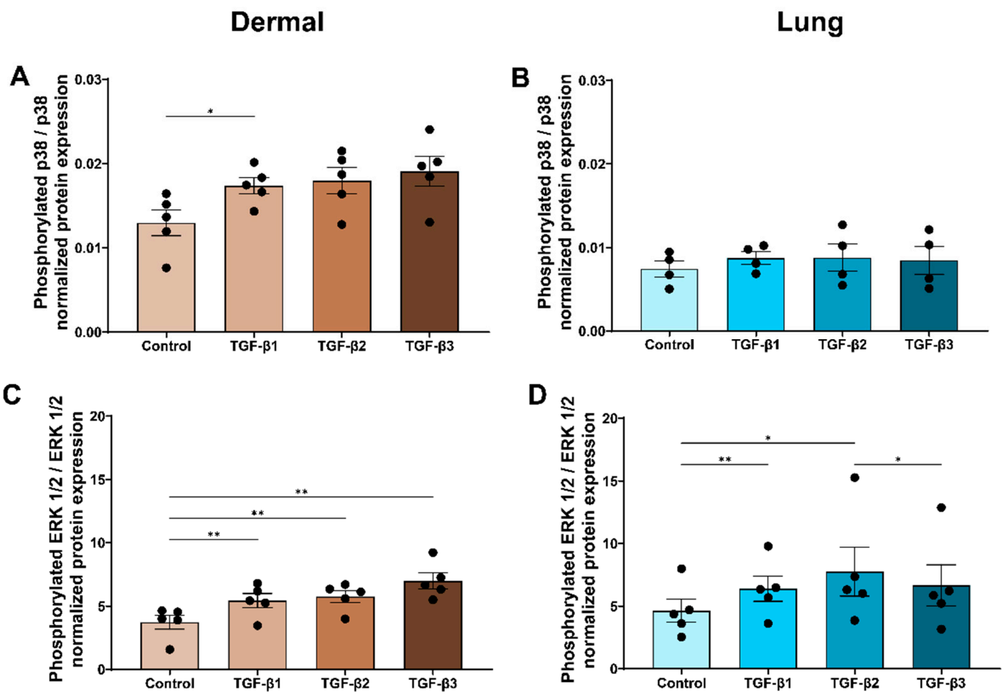

Supplementary Figure S5. Non-canonical TGF-β pathways, p38 and ERK1/2, are induced similarly by the TGF-β isoforms in dermal and lung fibroblasts when not adjusting for multiple comparisons. Dermal and lung fibroblasts (n = 5 biological replicates per cell line) were seeded in 6-well plates and stimulated with TGF-β isoforms 1, 2, and 3 for 6 h. Protein lysates were collected and analyzed using Western blotting to measure expression of p38 (**A–B**) and ERK1/2 (**C–D**). Data represent the mean with SEM. The Fisher's LSD test was used to test differences between treatment groups without correcting for multiple comparisons. *($p<0.05$), **($p<0.01$).
